# Parapharyngeal fat pad area at the subglosso-supraglottic level is associated with corresponding lateral wall collapse and apnea-hypopnea index in patients with obstructive sleep apnea: a pilot study

**DOI:** 10.1038/s41598-019-53515-5

**Published:** 2019-11-27

**Authors:** Hung-Chin Chen, Chao-Jan Wang, Yu-Lun Lo, Hao-Chun Hsu, Chung-Guei Huang, I-Chun Kuo, Yi-An Lu, Li-Jen Hsin, Wan-Ni Lin, Tuan-Jen Fang, Hsueh-Yu Li, Li-Ang Lee

**Affiliations:** 1 Department of Otorhinolaryngology-Head and Neck Surgery, Sleep Center, Linkou Chang Gung Memorial Hospital, Taoyuan, 33305 Taiwan, ROC; 2 Faculty of Medicine, College of Medicine, Chang Gung University, Taoyuan, 33302 Taiwan, ROC; 3 Department of Medical Imaging and Intervention, Sleep Center, Linkou Chang Gung Memorial Hospital, Taoyuan, 33305 Taiwan, ROC; 4 Department of Thoracic Medicine, Sleep Center, Linkou Chang Gung Memorial Hospital, Taoyuan, 33305 Taiwan, ROC; 5 Department of Bio-Industrial Mechatronics Engineering, National Taiwan University, Taipei, 10617 Taiwan, ROC; 6 Department of Laboratory Medicine, Linkou Chang Gung Memorial Hospital, Taoyuan, 33305 Taiwan, ROC; 7 Department of Medical Biotechnology and Laboratory Science, Graduate Institute of Biomedical Sciences, Chang Gung University, Taoyuan, 33302 Taiwan, ROC

**Keywords:** Health care, Medical research, Risk factors

## Abstract

The aim of this study was to assess associations between fat pad areas at various anatomic levels and the sites of lateral wall collapse and disease severity in adult patients with obstructive sleep apnea (OSA). Forty-one patients with OSA who prospectively underwent drug-induced sleep computed tomography were included. Areas of parapharyngeal fat pads and degrees of lateral wall collapse at three representative anatomic levels (nasopharynx, oropharynx, and subglosso-supraglottis), and apnea-hypopnea index (AHI) were measured. In the subglosso-supraglottic region, the parapharyngeal fat pad area in 17 (41%) patients with complete lateral wall collapse was significantly larger than that in 24 (59%) patients without complete collapse (median, 236.0 mm^2^ vs 153.0 mm^2^; P = 0.02). In multivariate regression analysis, the parapharyngeal fat pad area at the subglosso-supraglottic level (β = 0.02; P = 0.01) and body mass index (β = 3.24; P = 0.01) were independently associated with AHI. Our preliminary results supported that parapharyngeal fat pads at the subglosso-supraglottic level may be involved in the development of lateral wall collapse and then determine the severity of OSA. Further studies are warranted to investigate the effect of reducing parapharyngeal fat pads in the treatment of OSA.

## Introduction

Obstructive sleep apnea (OSA) is characterized by repeated partial or complete collapse of the upper airway during sleep contributing to intermittent hypoxia and hypercapnia^1,2^. Untreated OSA is associated with increased morbidity and mortality^3,4^. The etiology and pathophysiology of OSA are considered to be complex and multifactorial. Obesity is a known risk factor for the development and progression of OSA, however the mechanism is not fully understood. Fat deposits around the chest and abdomen reduce chest compliance and functional residual capacity, and increase oxygen demand^5^. Excessive soft-tissue surrounding the upper airway appears to result in a narrow and collapsible airway^6^. However, the effects of parapharyngeal fat pads at different levels on the severity of OSA are unclear.

Novel assessments of upper airway collapse are generally implemented during drug-induced sleep. Drug-induced sleep endoscopy provides tridimensional and dynamic evaluations of upper airway collapse under conditions best approximating natural sleep^7^. Although transverse retropalatal collapsibility, diagnosed using awake nasopharyngoscopy with Müller’s maneuver, is associated with the severity of OSA^8^, oropharyngeal and laryngo-hypopharyngeal collapses detected during wakefulness are frequently inconsistent to those detected during sleep^9^. Drug-induced sleep computed tomography (CT) can further detect subtypes of palate and tongue collapses using dynamic sagittal and coronal images, and document the intra-/extra-lumen airway structures using axial images^10^. In this study, we hypothesized that parapharyngeal fat pad areas at baseline may contribute to lateral wall collapse in drug-induced sleep which then exacerbates OSA.

The aim of this study, therefore, was to investigate the effects of parapharyngeal fat pad areas at various lateral wall levels on the sites of lateral wall collapse and disease severity in patients with OSA.

## Methods

This prospective cohort study was approved by the Institutional Review Board of the Chang Gung Memorial Foundation (No. 101-3547A3 and 102-5609A3). All procedures were carried out in accordance with the current regulations. Written informed consent was obtained from all participants.

The study recruited 41 adult patients who were diagnosed OSA by polysomnography at the Department of Otorhinolaryngology Head and Neck Surgery in Linkou Chang Gung Memorial Hospital (Taoyuan, Taiwan) between May 2013 and October 2015. All of the patients were either intolerant or unwilling to use long term continuous positive airway pressure therapy and sought to undergo intrapharyngeal upper airway surgery for OSA^10,11^. A dynamic drug-induced sleep CT was arranged in order to apply adequate surgical techniques to each patient based on his/her anatomic structure and collapse area^10,12^.

Anthropological parameters such as body mass index, neck circumference, tonsil grade, and Friedman tongue position were recorded^13^. Thirty-five patients with moderate-to-severe OSA have been reported previously^10,12^. The inclusion criteria were: (1) age 18–60 years; and (2) apnea-hypopnea index (AHI) >5. The exclusion criteria were: (1) allergy to propofol or a history of seizures; (2) pregnant or lactating women; (3) poor general condition for surgery; (4) altered upper airway anatomy due to previous airway or head and neck surgery; and (5) working abroad or incapable of regular follow-up^10,12^.

### Polysomnography

Sleep and breathing were documented by level I PSG (Nicolet UltraSom System, Madison, Wisconsin, USA). An apnoeic episode was defined as a drop in the peak thermal sensor excursion by at least 90% of baseline for at least 10 seconds. Hypopnea was defined as a decrease in airflow ≥30% with arousal or oxygen desaturation ≥4%^14^. The AHI was defined as the number of total apneic and hypopneic episodes per hour of sleep.

### Drug-induced sleep CT

All of the participants underwent a low-dose 320-detector row CT scan (Aquilion One dynamic volume CT system, Toshiba, Japan) using a consistent methodology that has been described previously^10,12^. The dynamic scans began from the orbital floor to the hyoid bone and continued for 10 seconds spanning 2–3 respiration cycles including inspirations and expirations^10^ with the patients awake and with propofol-induced light-sedation, respectively. Raw data were reconstructed and transferred to a workstation for post-processing, including axial, mid-sagittal, and coronal images and dynamic display. Pictures were uploaded and stored on a Picture Archiving and Communication System (Centricity Enterprise Web V3.0.10; GE Healthcare, Chalfont, UK).

### Anatomical identification at three parapharyngeal fat pad levels

**Figure 1:**
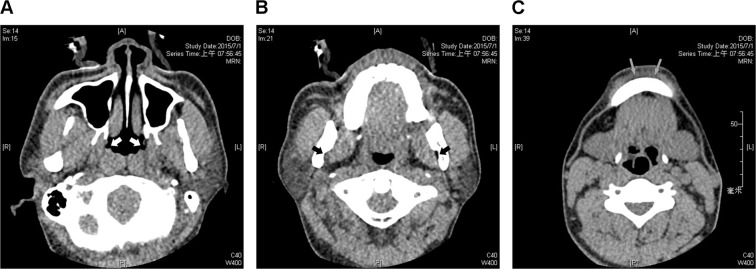
Radiographic landmarks of three representative parapharyngeal fat pad areas in axial images. The nasopharyngeal level of parapharyngeal fat pad was defined as where the Eustachian tube orifices (white arrows) are located (**A**). The oropharyngeal level was defined as where the mandibular foramens (black arrows) are located (**B**). The subglosso-supraglottic level was defined as where the mental tubercles (grey arrows) are located (**C**).

Three lateral wall levels (nasopharynx, oropharynx, and subglosso-supraglottis) were further defined by radiographic landmarks that could be easily recognized in axial CT images. The nasopharyngeal level was defined as where the Eustachian tube orifice was located (Fig. 1A). The oropharyngeal level was defined as where the mandibular foramen was located (Fig. 1B), and the subglosso-supraglottic level was defined as where the mental tubercle was located (Fig. 1C).

### Identification of the sites of lateral wall collapse

**Figure 2:**
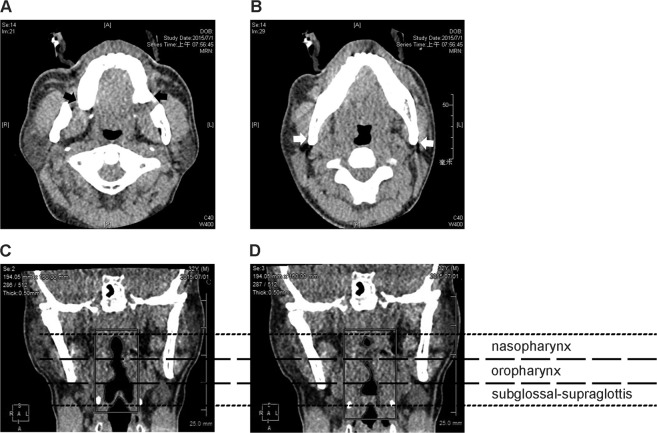
Radiographic landmarks of the parapharynx. The maxillary tuberosities (black arrows) were used as the landmarks of the retropalatal plane (**A**), and the mandible angles (white arrows) were used as the landmark of the retroglossal plane (**B**) in axial images. Sites of parapharyngeal lateral wall collapse were rated by comparing the maximal lateral wall length (**C**) and the minimal lateral wall length (**D**) of the nasopharynx, oropharynx, and subglosso-supraglottis during an apneic/hypopneic event in coronal images. In this representative patient, complete nasopharyngeal lateral wall collapse, complete oropharyngeal lateral wall collapse, and incomplete subglosso-supraglottic lateral wall collapse were detected.

Using the dynamic display of consecutive coronal views during drug-induced sleep, the lateral wall collapse (including the site(s) and severity) for each patient was rated by a single investigator as described previously^10,12^. To better characterize the sites of lateral wall collapse, two key radiographic landmarks of the axial views were used to define the classical anatomic planes: (1) the maxillary tuberosity for the retropalatal plane (Fig. 2A); and (2) the mandible angle for the retroglossal plane (Fig. 2B). Accordingly, “complete nasopharyngeal lateral wall collapse” was defined as “collapsibility ≥90% of the lateral wall above the maxillary tuberosity”. “Complete oropharyngeal lateral wall collapse” was defined as “collapsibility ≥90% of the lateral wall below the maxillary tuberosity and above the mandible angle”, and “complete subglosso-supraglottic lateral wall collapse” was defined as “collapsibility ≥90% of the lateral wall below the mandible angle”. For example, Fig. 2C,D demonstrate a representative patient with complete nasopharyngeal collapse, complete oropharyngeal collapse, and incomplete subglosso-supraglottic collapse.

### Measurement of the pharyngeal fat pad areas

All axial CT images with a proportional scale with the patients awake were captured in Joint Photographic Experts Group format without modifying the image quality. We used the image region analyzer application running on the MATLAB platform (MathWorks, Natick, MA, USA) to measure the parapharyngeal fat pad areas. Ten points of the supposed parapharyngeal fat pad were randomly chosen by an experienced investigator (who was blind to the polysomnographic data). The mean intensity and SD were automatically calculated using the original pixel values. The areas where the original pixel value was lower than “mean + 1 × SD” or “mean + 2 × SD” were marked and then processed using morphological opening with a two-pixel boundary. The area in pixels was finally converted to mm^2^ using a proportional scale.

### Validation of parapharyngeal fat pad measurement

**Figure 3:**
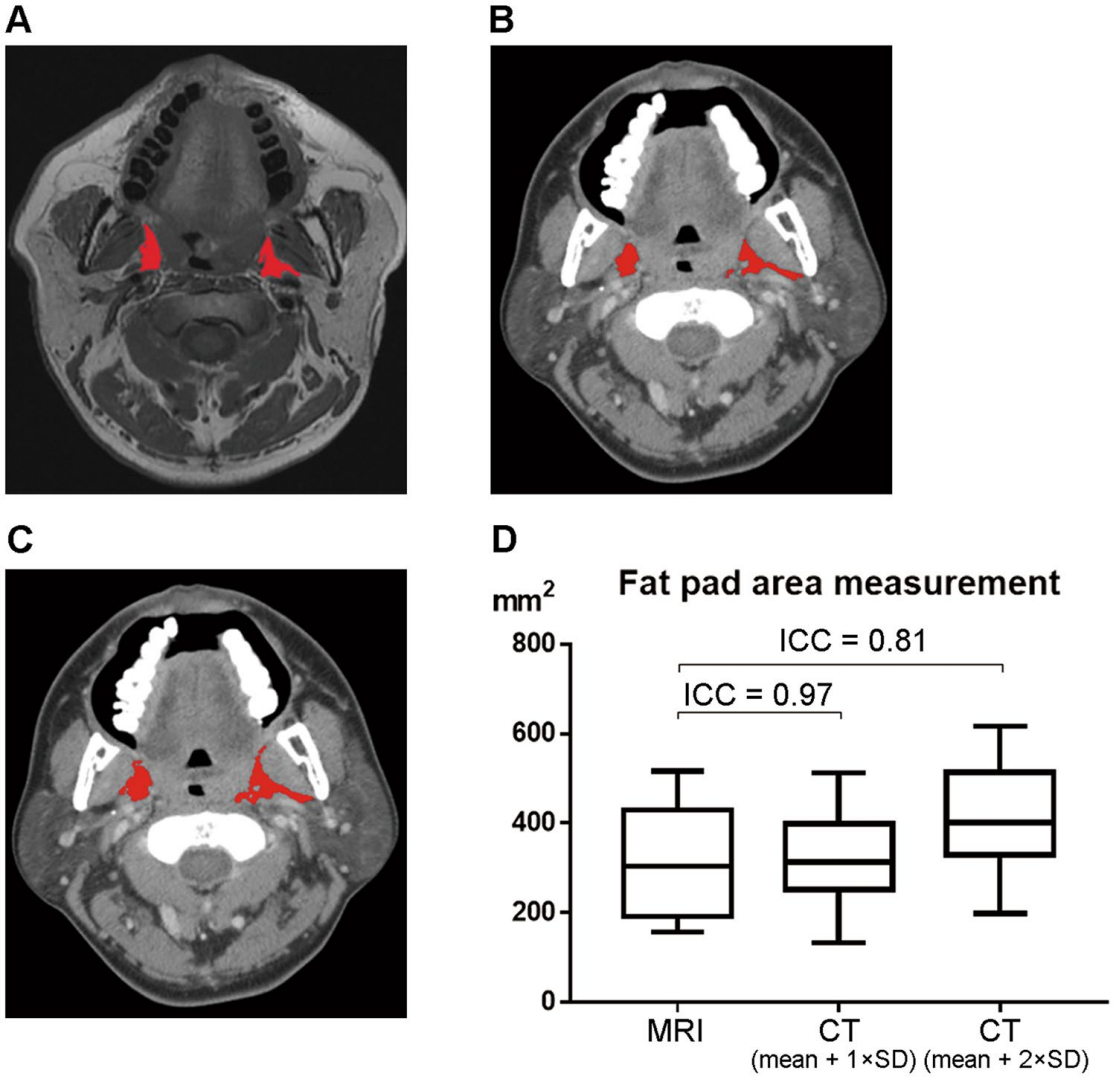
Validation of parapharyngeal fat pad area measurement by CT. Representative parapharyngeal fat pad areas (red areas) at the oropharyngeal level are labeled according the criterion of “the area below mean + 1 × SD original pixel value” in an axial MRI image (**A**). Representative parapharyngeal fat pad areas (red areas) at the same level in the same patient are labeled according to the criteria “the area below mean + 1 × SD original pixel value” (**B**) and “the area below mean + 2 × SD original pixel value” (**C**) in an axial CT image. The intraclass correlation coefficient (ICC) of pharyngeal fat pad areas detected by MRI and those detected by CT using the criterion of “mean + 1 × SD” was higher than that of pharyngeal fat pad areas detected by MRI and those detected by CT using the criterion of “mean + 2 × SD”.

We randomly selected four other patients who underwent both CT and magnetic resonance imaging (MRI) of the head and neck due to oral cancer at our department in the pre-study. CT and MRI scans of the nasopharynx, oropharynx and subglosso-supraglottis at the same level were obtained. Therefore, a total of 12 images were analyzed. The parapharyngeal fat pad areas were calculated using the criteria of “mean + 1 × SD” or “mean + 2 × SD” by MRI. The average parapharyngeal fat pad areas were similar (P > 0.05; data not shown). Accordingly, the mean (SD) parapharyngeal fat pad area by MRI using the criterion of “mean + 1 × SD” was 310.4 mm^2^ (124.8 mm^2^) and was used as a reference value (Fig. 3A). The mean (SD) parapharyngeal fat pad areas calculated by CT using the criterion of “mean + 1 × SD (Fig. 3B)” and “mean + 2 × SD (Fig. 3C)” were 322.36 mm^2^ (110.59 mm^2^) and 424.31 mm^2^ (129.33 mm^2^), respectively. The intraclass correlation coefficient between CT measurements using the criterion of “mean + 1 × SD” and MRI measurements was 0.97, and the intraclass correlation coefficient between CT measurements using the criterion of “mean + 2 × SD” and MRI measurements was 0.81 (Fig. 3D). To avoid over-estimation, we calculated the parapharyngeal fat pad area using the criterion of “mean + 1 × SD original pixel value” in CT images. Measurements were made by the same investigator 2 weeks later. The intra-rater reliability of measurements of the nasopharyngeal, oropharyngeal and subglosso-supraglottic pharyngeal fat pad areas were 0.90, 0.95 and 0.92, respectively.

### Measurement of the mandibular plane-hyoid distance

**Figure 4:**
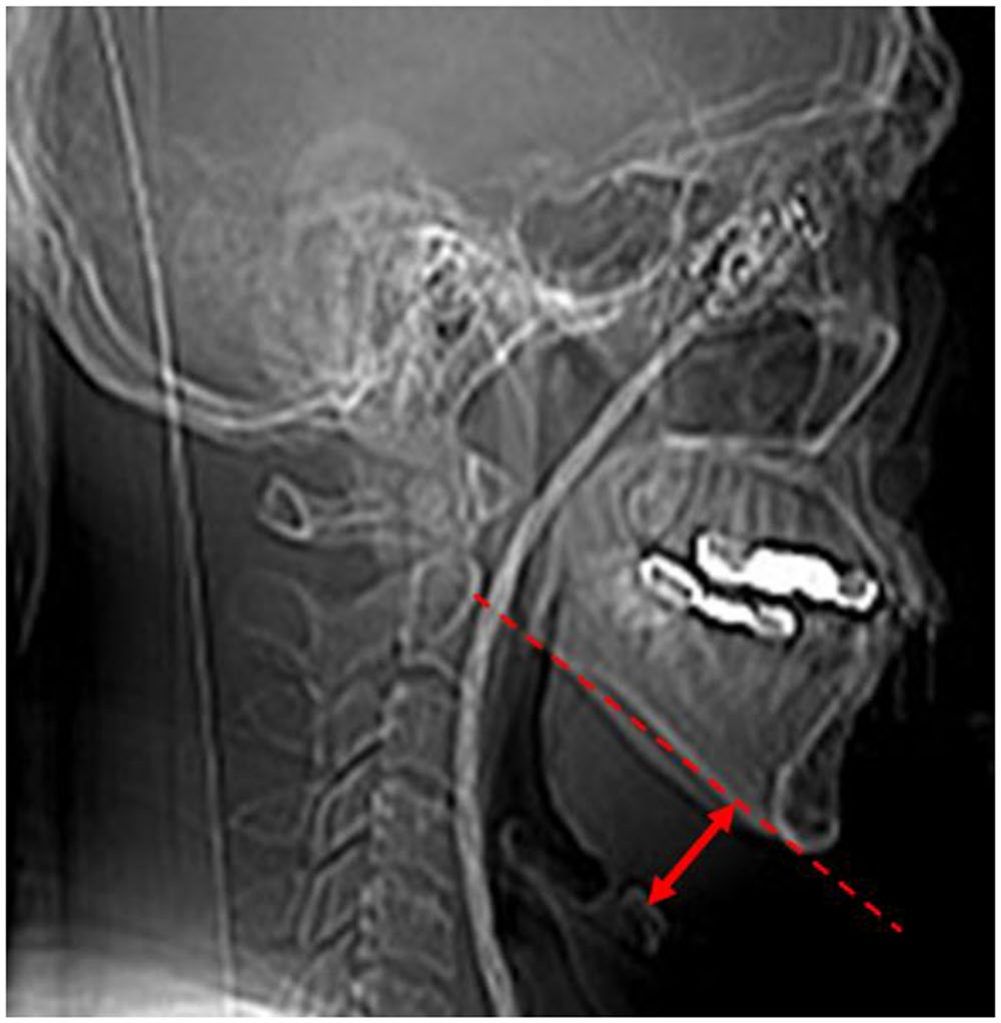
The mandible plane-hyoid distance (MPH) measurement. The MPH (red double arrow) was defined as the vertical distance from mandibular plane (red dotted line) to hyoid bone using a plain film of a computed tomography scan.

The distance from mandibular plane to hyoid bone was defined as ‘mandibular plane-hyoid distance (MPH)’ in lateral cephalometric analysis^15,16^. In this study, we utilized plain film of our CT series to take a measurement of the MPH (Fig. 4).

### Statistical analysis

The Kolmogorov-Smirnov normality test showed that most distributions of the variables were non-normal, and data were reported as median and interquartile range or number and frequency, as appropriate. Parameters were compared using the Mann-Whitney U test, Kruskal-Wallis test, Wilcoxon signed rank test, chi-square test, or Fisher’s exact test, as appropriate. The Spearman correlation test was used to analyze relationships between variables. Variables with a P value < 0.05 in the Spearman nonparametric correlation test were included in multivariate linear regression analysis. Two-tailed P-values < 0.05 were considered to be statistically significant. Statistical analyses were conducted using SPSS software version 23 (International Business Machines Corp., Armonk, NY, USA).

## Results

### Study population

Table 1 summarizes the demographic characteristics, AHI, and parapharyngeal fat pad areas of the subjects. Thirty-nine (95%) men and two (5%) women with a median age of 40 years, median body mass index of 26.5 kg/m^2^, and median AHI of 50.2 events/h were included.

**Table 1:** Patient demographics, apnea-hypopnea index, and parapharyngeal fat pad areas in patients with obstructive sleep apnea.

Variables	n = 41
Age, years	40 (34–48)
Male gender	39 (95%)
Body mass index, kg/m^2^	26.5 (24.5–28.3)
Normal weight (<25)	13 (32%)
Overweight (25–30)	22 (54%)
Obesity (>30)	6 (14%)
Neck circumference, cm	39.5 (37.8–40.8)
Tonsil grade III–IV	8 (20%)
Tongue position III–IV	21 (51%)
MPH^a^, mm	22.4 (12.5–33.5)
Apnea-hypopnea index, event/h	50.2 (32.2–65.80)
Mild (range, 5–15)	6 (15%)
Moderate (range, 16–30)	4 (10%)
Severe (range, ≥30)	31 (75%)
**Site of compete lateral wall collapse**^**b**^	
Nasopharynx	2 (4.9%)
Oropharynx	24 (58.5%)
Subglosso-supraglottis	17 (41.5%)
**Parapharyngeal fat pad area**	
Nasopharyngeal level, mm^2^	347.6 (241.8–593.8)
Oropharyngeal level, mm^2^	322.4 (202.8–605.8)
Subglosso-supraglottic level, mm^2^	168.0 (118.3–241.1)

All values are reported as median and interquartile range or number (percentage), as indicated.

^a^MPH: Mandibular plane-hyoid distance.

^b^Patients might have more than or equal to one site of complete lateral wall collapse that collapsibility was more than 90%.

### Sites of complete lateral wall collapse

Ttwo subjects (5%) had complete nasopharyngeal lateral wall collapse, 24 (59%) had complete oropharyngeal lateral wall collapse, and 17 (42%) had complete subglosso-supraglottic lateral wall collapse (Table 1).

### Parapharyngeal fat pad area

The median parapharyngeal fat pad area at the nasopharyngeal level was equivalent to that at the oropharyngeal level, whereas the median parapharyngeal fat pad area at the subglosso-supraglottic level was significantly lower than that at other levels (P < 0.001) (Table 1).

### Correlation of parapharyngeal fat pad areas and MPH with sites of complete lateral wall collapse

Table 2 demonstrates the relationships between parapharyngeal fat pad areas and sites of complete lateral wall collapse. The parapharyngeal fat pad areas at the nasopharynx and oropharynx were not significantly correlated with complete nasopharyngeal and oropharyngeal lateral wall collapses. Of note, the parapharyngeal fat pad areas at the subglosso-supraglottic level were significantly correlated with complete subglosso-supraglottic lateral wall collapse (r = 0.36; P = 0.02). At the subglosso-supraglottic level, the median parapharyngeal fat pad area (median, 236.0 mm^2^; interquartile range, 124.8–334.8 mm^2^) in the patients with complete lateral wall collapse was significantly larger than that (median, 153.0 mm^2^; interquartile range, 112.6–179.1 mm^2^) in the patients without complete collapse (P = 0.02). The MPH was related to BMI (r = 0.41; P = 0.01) but not associated with lateral pharyngeal collapse (Table 2).

**Table 2:** Relationships between parapharyngeal fat pad areas, mandibular plane-hyoid distance and sites of complete lateral wall collapse and other variables.

Variables	Parapharyngeal fat pad area			MPH^b^
	Nasopharyngeal level	Oropharyngeal level	SG-SG^a^ level	
Age	−0.028 (0.864)	−0.090 (0.576)	0.085 (0.599)	−0.074 (0.646)
Male gender	−0.019 (0.905)	<0.001 (1.000)	−0.038 (0.812)	0.077 (0.634)
Body mass index	0.147 (0.358)	0.137 (0.395)	0.241 (0.128)	0.410 (0.008*)
Neck circumference	0.147 (0.360)	0.067 (0.678)	0.268 (0.091)	0.180 (0.260)
Tonsil grade	0.290 (0.066)	0.242 (0.127)	0.389 (0.012*)	0.042 (0.794)
Tongue position	0.098 (0.542)	0.077 (0.630)	0.170 (0.287)	0.246 (0.121)
**Site of compete lateral wall collapse**^**c**^				
Nasopharynx	0.172 (0.282)	0.144 (0.371)	0.067 (0.677)	0.057 (0.721)
Oropharynx	0.155 (0.334)	0.067 (0.678)	0.046 (0.775)	0.172 (0.284)
Subglosso-supraglottis	0.268 (0.091)	0.197 (0.218)	0.360 (0.021*)	0.268 (0.091)

All values are reported as Spearman correlation coefficient (*P*-value).

^a^SG-SG: Subglosso-supraglottis.

^b^MPH: Mandibular plane-hyoid distance.

^c^Patients might have more than or equal to one site of complete lateral wall collapse that collapsibility was more than 90%.

*Two-tailed *P*-values less than 0.05.

### Relationship between parapharyngeal fat pad areas and AHI

Body mass index, neck circumference, tonsil grade, parapharyngeal fat pad area at the nasopharyngeal level, and parapharyngeal fat pad area at the subglosso-supraglottic level were significantly associated with AHI (Table 3). In multivariate analysis, parapharyngeal fat pad area at the subglosso-supraglottic level (β = 0.07; 95% confidence interval, 0.02–0.11; P = 0.01) and body mass index (β = 3.24; 95% confidence interval, 0.79–5.69; P = 0.01) were independent predictors of AHI.

**Table 3:** Univariate and multivariate analyses of apnea-hypopnea index with parapharyngeal fat pad areas and other variables.

Variables	Univariate Spearman correlation test		Multivariable linear regression test	
	coefficient	*P*-value	β (95% CI)	*P*-value
Age	−0.06	0.71	—	NI
Male gender	0.01	0.95	—	NI
Body mass index	0.37	0.02*	3.24 (0.79–5.69)	0.01*
Neck circumference	0.32	0.045*	—	NS
Tonsil grade	0.37	0.02*	—	NS
Tongue position	0.27	0.08	—	NI
MPH^a^	0.29	0.07	—	NI
**Site of compete lateral wall collapse**^**b**^				
Nasopharynx	−0.12	0.44	—	NI
Oropharynx	0.14	0.39	—	NI
Subglosso-supraglottis	0.334	0.03*	—	NI
**Parapharyngeal fat pad area**				
Nasopharyngeal level	0.35	0.02*	—	NS
Oropharyngeal level	0.18	0.26	—	NI
Subglosso-supraglottic level	0.63	<0.001*	0.07 (0.02–0.11)	0.01*

Abbreviations: CI, confidence interval; NI, not included; NS, not significant.

^a^MPH: mandibular plane-hyoid distance.

^b^Patients might have more than or equal to 1 site of complete lateral wall collapse that collapsibility was more than 90%.

*Two-tailed *P*-values less than 0.05.

## Discussion

In this pilot study, we developed a program to measure parapharyngeal fat pad areas using images of drug-induced sleep CT, a well-established tool used to demonstrate dynamic changes in the upper airway during simulated sleep^10^. We found significant associations between parapharyngeal fat pad area at the subglosso-supraglottic level, subglosso-supraglottic lateral wall collapse, and AHI. That is, increased parapharyngeal fat pad area at the subglosso-supraglottic level could induce corresponding lateral wall collapse and further exacerbate the severity of OSA.

Quantifying the effects of parapharyngeal fat tissue on upper airway collapse is crucial to understand the competing biomechanical processes that maintain airway patency^3^. Using passive critical closing pressure to assess the anatomic component predisposing to OSA, tongue volume and neck circumference have been associated with upper airway collapsibility^17^, although soft tissue enlargement does not always contribute to upper airway collapse^18^. Nevertheless, there is currently no consensus on anatomical reference points/levels of the pharyngeal region to measure parapharyngeal fat pad areas. For example, the internal fat volume of the retroglossal neck has been associated with the lateral width of the smallest cross-section of the retropalatal airway^19^. In addition, an increase in retropalatal/retroglossal parapharyngeal fat pad volume has been associated with an increased likelihood of representing the concentric type of retropalatal collapse determined by drug-induced sleep endoscopy^2^. To improve consistency and make communication clearer, we established a systematic approach to measure parapharyngeal fat pad areas including two axial planes and three anatomic levels according to five definite radiographic landmarks. In our preliminary results, parapharyngeal fat pad areas were not associated with lateral wall collapse at the corresponding level except for the subglosso-supraglottic level. We hypothesize that the effect of parapharyngeal fat pads contributing to airway collapse may be attenuated at the nasopharyngeal and oropharyngeal levels where bulky and thick soft tissues (soft palate, tonsils, and tongue) mainly obstruct the upper airway. In contrast, no considerable obstructive soft tissues were found in the subglosso-supraglottic region, and increased parapharyngeal fat pads may have enhanced lateral wall collapse.

Furthermore, the influence of parapharyngeal fat pads on the development of OSA is still under debate. For example, patients with OSA have been shown to have more parapharyngeal fat tissue than body mass index-matched controls in MRI^20^. However, another study showed that neck visceral fat volume was not significantly associated with AHI in very obese patients^21^. In addition, CT studies have suggested that pharyngeal fat tissue and neck fat distribution seem to play an important role in the development of early stage OSA in obese patients^22^ and exacerbate the severity of OSA in men with abdominal obesity undergoing growth hormone treatment^23^. However, another study reported no significant association between retropalatal/retroglossal parapharyngeal fat pad volume and AHI^2^. We found that only the parapharyngeal fat pad area at the subglosso-supraglottic level was correlated with AHI. We hypothesize that parapharyngeal fat pads at this level may exacerbate the severity of OSA by increasing lateral wall collapsibility.

The MPH value was reported higher in patients with OSA, especially in those who had BMI more than 25 kg/m^2^, as compared to habitual snorers^24,25^. The MPH assessed by cephalometry was positively correlated with AHI and tongue obstruction^16,26,27^. In our study, we found the MPH was positively related to BMI, and tended to be positively associated with the AHI. The possible reasons include: (1) We measured the MPH using a plain film of a three-dimensional CT scan (not a standard cephalometry) which might contribute a methodological bias; and (2) One-third of the subjects with BMI less than 25 kg/m^2^ might confound the association between the MPH and AHI.

Obesity and particularly central adiposity can alter upper airway anatomy and neuromuscular control and increase susceptibility to OSA^28^. As the STOP-BANG questionnaire includes body mass index and neck circumference, it has consistently shown high sensitivity for detecting OSA of various severity^29^. In this study, we found that a novel parameter, subglosso-supraglottic parapharyngeal fat pad area, which is independent of both body mass index and neck circumference, influenced the severity of OSA. However, further studies are needed to verify the clinically significant threshold of the subglosso-supraglottic parapharyngeal fat pad area.

A significant increase in the proportion of severe subglosso-supraglottic lateral wall collapse between patients with surgical failure and success has been reported^30^. Although many surgical techniques can achieve soft tissue reduction and reposition^31^, no current OSA surgery targets parapharyngeal fat pads. Of note, an increase in parapharyngeal fat pad in the subglosso-supraglottic region may contribute to lateral wall collapse, thereby leading to surgical failure. Therefore, further studies are warranted to investigate the effect of reducing parapharyngeal fat pads in the treatment of OSA.

There are several limitations to this study. First, three quarters of the patients met the criteria of severe OSA, and almost all were male. Further studies are needed to elucidate whether our results can be applied to patients with mild OSA or females. Second, we used subjective assessments of complete lateral wall collapse in this pilot study. Further studies are needed to objectively quantify airway collapsibility such as with critical closing pressure. Third, we did not include standard cephalometric assessment to demonstrate whether mandible or maxilla retrusion influence pharyngeal collapse and surgical results^26^. Fourth, it would be ideal to include a control group without OSA. Further prospective large-scale studies will be helpful to elucidate these related topics.
